# Study on the Influencing Factors of UHPC Durability and Its Microscopic Performance Characterization

**DOI:** 10.3390/ma18143268

**Published:** 2025-07-10

**Authors:** Risheng Wang, Yongzhuang Zhang, Hongrui Wu, Xueni Jiang

**Affiliations:** School of Transportation and Civil Engineering, Shandong Jiaotong University, Jinan 250357, China; 18369249197@163.com (Y.Z.); hongruiw0925@163.com (H.W.); 19025311124@163.com (X.J.)

**Keywords:** mix ratio, curing method, aggregate, admixture, corrosion characteristics, durability

## Abstract

Considering the harsh marine environment characterized by dry–wet cycles, freeze–thaw action, chloride penetration, and sulfate attack, four optimized ultra-high-performance concrete (UHPC) mix designs were developed. Durability was assessed via electric flux, dry–wet cycles, and rapid freeze–thaw tests to evaluate the effects of curing methods, aggregate types, and mineral admixtures on key durability indicators, including chloride ion permeability, compressive strength loss, and mass loss. Scanning electron microscopy (SEM) examined microstructural changes under various conditions. Results showed that curing method significantly affected chloride ion permeability and sulfate resistance. High-temperature curing (70 ± 2 °C) reduced 28-day chloride ion electric flux by about 50%, and the compressive strength loss rate of specimens subjected to sulfate attack decreased by 2.7% to 45.7% compared to standard curing. Aggregate type had minimal impact on corrosion resistance, while mineral admixtures improved durability more effectively. Frost resistance was excellent, with mass loss below 0.87% after 500 freeze–thaw cycles. SEM analysis revealed that high-temperature curing decreased free cement particles, and mineral admixtures refined pore structure, enhancing matrix compactness. Among all mixtures, Mix Proportion 4 demonstrated the best overall durability. This study offers valuable insights for UHPC design in aggressive marine conditions.

## 1. Introduction

As global environmental challenges intensify, the construction of sustainable and resilient infrastructure systems has become increasingly urgent. As emphasized by Professor Raymondt [1] of the University of Oxford, “Let’s be honest and blunt. About the climate crisis—yes, it’s time to panic”. This stark warning highlights the pressing need to address both the environmental impact and long-term durability of building materials, particularly concrete, which is resource-intensive and highly susceptible to degradation in marine environments. Against the backdrop of China’s national strategy to become a maritime power, offshore infrastructure is developing rapidly. According to the China Marine Economy Statistical Bulletin (2024) [2], the marine engineering construction industry in China achieved an added value of 219.4 billion CNY in 2024. As major infrastructure projects such as cross-sea bridges, offshore oil and gas platforms, and deep-water ports continue to advance, the demand for high-performance construction materials is steadily increasing. In these projects, concrete serves as a key structural material and faces severe durability challenges in marine environments, which directly impact its service life and environmental performance. To date, numerous international studies have highlighted the close relationship between environmental sustainability and concrete durability. For instance, Mehta and Monteiro emphasized that excessive cement use significantly increases CO_2_ emissions, and enhancing concrete durability contributes to reducing its life-cycle environmental impact [3]. Furthermore, Scrivener et al. stressed that optimizing offshore concrete materials is crucial for achieving both extended structural service life and sustainable development [4]. Therefore, improving the durability of concrete through optimized mix design, curing conditions, and material selection is a critical pathway to enhancing resource utilization efficiency and achieving sustainable marine infrastructure construction.

Ultra-High-Performance Concrete (UHPC), with its exceptional mechanical properties and outstanding durability, has emerged as a highly promising solution [5]. Pierre Richard [6] was among the first to systematically study UHPC, laying the foundation for its application in structural engineering. Subsequent studies [7] have confirmed UHPC’s superior resistance to chloride penetration, sulfate attack, and mechanical degradation.

Regarding curing conditions [8], Sun, Zhang, Mao, et al. [9] confirmed that appropriate temperature and humidity environments can effectively improve the microstructure of UHPC. Li Mao et al. [10] found that excessively high steam curing temperatures lead to a three-stage evolution of freeze–thaw damage in concrete. In their study, the cumulative number of acoustic emission events was used as an indirect and quantitative indicator to assess the extent of internal damage development and structural deterioration. They further confirmed the cumulative nature of internal damage through acoustic emission monitoring techniques. Davraz et al. [11], using response surface methodology, optimized the curing process and found that steam curing at 60 °C balances compressive strength with cost efficiency. Although setting accelerators can improve early strength, they significantly increase the cost.

In terms of the application of mineral admixtures, Sudarsono et al. [12] demonstrated that partial replacement of cement with fly ash significantly improves the chloride ion resistance of concrete, although its carbonation resistance remains relatively limited. To overcome the performance limitations of single admixtures, Maohua et al. [13] reported that the combined use of fly ash and silica fume can not only enhance compressive strength but also significantly improve resistance to chloride penetration. This synergistic effect was further confirmed by Raj Kishore, G.V.V.K. through experimental investigations [14]. Wang et al. [15] showed that under dry–wet cycles, concrete with 30% slag exhibits superior sulfate resistance compared to an equivalent amount of fly ash, and the inclusion of silica fume effectively improves freeze–thaw resistance by reducing pore volume and pore fractal dimension. Zhao Yaming et al. [16] found that replacing silica fume with fly ash or slag reduces the proportion of fine pores, increases average pore diameter, and mitigates autogenous shrinkage. Moreover, Singh et al. [17] partially replaced cement with metakaolin and silica fume while using copper slag as fine aggregate; their results indicated that a 50% replacement ratio of copper slag yielded the best durability performance under multiple aggressive conditions. Furthermore, Gu et al. [18] confirmed that the incorporation of silica fume densifies the microstructure of concrete during freeze–thaw cycles, thereby significantly enhancing frost resistance.

Despite significant progress, Abbas and Khan [19] pointed out that most studies still focus on single environmental factors, lacking systematic understanding of degradation mechanisms under coupled multi-factor conditions. Additionally, Ghafari et al. [20] found that incorporating nanosilica significantly enhances UHPC’s resistance to chloride and sulfate attack. Maohua Zhang, Lin Deng, Zhiyi Li, and Tiejun Sun [21,22,23,24] further demonstrated that various nanoparticles improve marine concrete durability under combined chloride erosion, carbonation, fatigue, and wet–dry cycles. Although nanomaterials offer excellent performance, their high cost and complex synthesis limit their application in large-scale projects. In contrast, mineral admixtures, as industrial by-products, are cost-effective and widely available. With proper design, they can also achieve performance enhancements comparable to nanomaterials. This comparative advantage underlines the potential of mineral admixtures as a cost-effective alternative to nanomaterials in UHPC design.

Based on the above, this study proposes four optimized mix designs of UHPC for marine applications. A continuous gradation of quartz sand in the ranges of 1.25–0.63 mm and 0.63–0.315 mm was selected, with a water-to-binder ratio (W/B) of 0.17 and a superplasticizer dosage of 1.0%. In Mixes 1 and 2, a sand-to-binder ratio of 1.2 was adopted, incorporating different types of quartz sand. Mixes 3 and 4 included mineral admixtures with a ratio of fly ash:silica fume:slag powder = 0.6:0.2:0.2. The proposed water-to-binder ratio of 0.17 and sand-to-binder ratio of 1.2, while slightly increasing the demand for superplasticizer, can be feasibly implemented in precast or factory-controlled marine construction scenarios. These environments allow precise quality control and uniform curing conditions, ensuring the consistency of low-W/B mixes. Moreover, by reducing the dosage of expensive silica fume and incorporating cost-effective fly ash and slag, the overall material cost is significantly reduced. When evaluated over the service life of marine structures, the enhanced durability leads to lower maintenance and repair costs, making the mix design economically viable for large-scale applications.

This study systematically investigates the chloride permeability, sulfate attack resistance, freeze-thaw performance, and microstructural characteristics of UHPC under varying sand types, curing temperatures, and mineral admixture conditions by optimizing the preparation process and mix design. Durability indices were evaluated using rapid chloride permeability tests, wet-dry cycle tests, and rapid freeze-thaw testing, while microstructure was examined through scanning electron microscopy (SEM) to reveal the coupling relationship between internal composition, morphology, and durability of UHPC. The results contribute to a deeper understanding of the degradation mechanisms of UHPC in complex marine environments and provide theoretical support for its material optimization and design.

## 2. Preparation of UHPC and Determination of the Reference Mix Proportion

### 2.1. Optimization of Concrete Mixing Process and Determination of Curing Method

#### 2.1.1. Mixing Process Optimization

**Figure 1:**
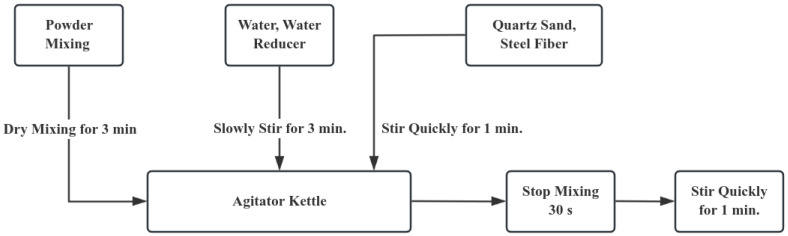
Preparation Technology.

According to Zhao, Huang, and Xie [25], the compressive strength of UHPC specimens prepared using three commonly adopted mixing processes was measured after 3 days of curing under standard conditions. The results show that the powder material should be mechanically stirred for 5 min first, and then the water reducing agent and water are added. Then, continue mechanical stirring for 8 min, add steel fibers, and finally keep stirring for 10 min. After that, keep on mechanically stirring for 8 min, put in the steel fibers, and at last, keep stirring continuously for 10 min. The UHPC specimens prepared by this process have the highest compressive strength. However, considering the limitations of the preparation conditions at the construction site, an excessively long mixing time may delay the construction progress. At the same time, long-term static placement will also lead to the segregation of the concrete. Therefore, based on the above process, this article optimizes the UHPC preparation process by adjusting the mixing time. The detailed preparation procedure is depicted in Figure 1:

After the mixing is completed, the specimens are formed by the layered vibration method. Firstly, pour the mixture into the mold until it reaches one-third of the mold’s height. Put it on the vibrating table and continuously vibrate it to make it dense. Meanwhile, carry out poking and ramming along the inner side of the test mold. Once again, keep adding the mixture until it reaches two-thirds of the height of the mold. Carry out vibration once more to make it dense. Lastly, replicate the aforementioned procedures until the mold is completely filled.

#### 2.1.2. Specimen Curing Protocol

Temperature regulation, which accelerates the hydration process and improves concrete performance, has been widely employed in concrete preparation, as supported by existing research [9]. Given the variability in environmental conditions and differences in material strength requirements, different types of concrete often necessitate distinct curing strategies.

To further investigate the impact of curing temperature on the overall performance of UHPC, this study employed two different curing methods for comparative analysis. Through comprehensive evaluation of the experimental results, the optimal curing parameters were identified.

The first curing method is the standard curing procedure. The specimens were first submerged in water to ensure full surface contact and then placed in a constant-temperature curing chamber maintained at (20 ± 2) °C. The curing durations were 3 days, 7 days, and 28 days, respectively. During the curing process, the water in the tank was regularly replaced to maintain a stable environment.

The second curing method involved high-temperature water bath curing. The specimens were placed in a temperature-controlled water bath tank, where the heating rate did not exceed 12 °C per hour. Once the water temperature reached 70 °C, the specimens were cured at a constant temperature of (70 ± 2) °C for the same durations of 3, 7, and 28 days.

### 2.2. Mixing Ratio Design

#### 2.2.1. Design Methodology

Studies have demonstrated that the compactness of particulate fillers plays a critical role in determining the mechanical properties and durability of cementitious composites. To enhance the particle packing structure of UHPC, several models have been developed. Among the most commonly used are the Andreasen model, the modified Andreasen model based on the Fuller curve, and the Dinger–Funk packing model, which was proposed by Dinger and Funk as an extension of the Andreasen model [26].

The Dinger–Funk packing model is considered more applicable to engineering practice because it comprehensively accounts for the actual packing behavior of particles and introduces a minimum particle size into the equation. Therefore, this study adopts the Dinger–Funk model for mixture proportion design, and the corresponding equation is given as follows.

(1) $$U(D)=\frac{D^{n}-D_{\min}^{n}}{D_{\max}^{n}-D_{\min}^{n}}\times 100\%$$

where U(D) is cumulative percentage passing the sieve (%); D is particle size (mm); $D_{\min}$ is minimum particle size (mm); $D_{\max}$ is Maximum particle size (mm); n is Distribution modulus.

Considering that the particles are relatively fine, the value of n is selected as 0.3.

#### 2.2.2. Design Objectives

To investigate the effects of various factors on the durability of UHPC and to determine the optimal mix proportions, this study adopts the provisions and dosage requirements specified in Reactive Powder Concrete (GB/T 31387-2015) [27] as the fundamental design guidelines. Based on these standards, the mix design of UHPC was formulated. The design principles governing the dosage ratio of each constituent material are summarized in Table 1.

The basic indexes of the mechanical and durability properties of UHPC are shown in Table 2 and Table 3. It should be pointed out that Reactive Powder Concrete” (GB/T 31387-2015) [27] “Basic Properties and Test Methods of Ultra-High Performance Concrete” (T/CBMF 37—2018/T/CCPA 7—2018) [28] fails to give a clear stipulation on the fluidity index of UHPC. To guarantee the successful and high-quality molding of the specimens, this research makes comprehensive reference to document [29,30] and incorporates numerous experimental validations. Ultimately, the minimum limiting value of the fluidity of the UHPC under study is ascertained as 160 mm.

#### 2.2.3. Quartz Sand Grading Selection

According to the literature [31], the results of the packing density and porosity of quartz sand under different gradations obtained by theoretical calculation are shown in Table 4.

By analyzing Table 4, it is found that under the single particle size grading, the packing density of quartz sand rises along with the augmentation of the maximum particle size, while the pore volume fraction gradually decreases. When Dmax increases from 0.315 mm to 1.25 mm, the bulk density of quartz sand increases by 6.6%, and the porosity decreases by 8.4%. Under the continuous gradation, the bulk density and porosity of quartz sand show the same variation trend. When Dmax increases from 0.63 mm to 1.25 mm, the bulk density of quartz sand increases by 3.9% year-on-year, and the porosity decreases by 5.8% year-on-year. However, from a horizontal comparison, when the maximum particle size is the same, the bulk density of the continuously graded quartz sand is greater, and the porosity is smaller. This is mainly because the quartz sand with secondary particle size plays a filling role, making the structure more compact. In view of this, in this study, two kinds of quartz sands with continuous gradations of 1.25–0.63 mm and 0.63–0.315 mm are selected for comparative analysis with the standard sand. The test findings show that when the ratio of sand to binding material of UHPC is greater than 1.3, its spread value drops sharply, and the spread value is the best when the proportion of sand to the binder is between 1.0 and 1.3.

#### 2.2.4. Trial Mixing of UHPC

Based on the established mix design equation, the component dosage regulations, the specified performance requirements, and the determined quartz sand gradation, cement, standard sand, quartz sand, mineral admixtures, water reducers, steel fibers, and water were selected as the test materials. Four sets of mix proportions were designed accordingly, as detailed in Table 5.

The fluidity test of the neat paste of UHPC was carried out, and the fluidity of the initial mix proportion is presented in Table 6.

**Figure 2:**
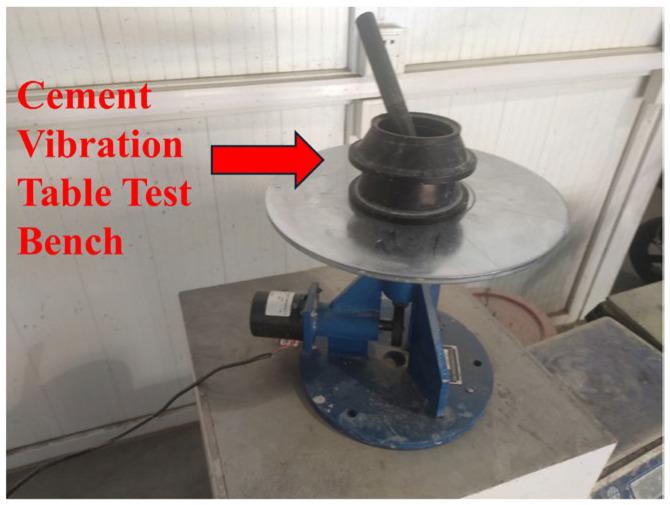
Cement jump table test bench.

Considering that the coarse aggregate is removed from UHPC, the flow table test [32,33,34] can be used to more quickly and intuitively observe the flow state of the sample and conduct a quantitative and standardized evaluation. Therefore, in this study, the flow table test was carried out on the mixture to determine its fluidity. The experimental equipment is shown in Figure 2.

In the test, the sample was put into the mold two times. First, the first layer of the sample was filled to two-thirds of the height of the mold, and then the sample was compacted 15 times with a tamping rod from the edge of the mold to the center. Then, the second layer of the sample was filled to a position slightly 20 mm higher than the mold, and compacted 10 times again to complete the sample filling. The mold was lifted vertically, and the flow table was started. The sample was vibrated 25 times at a frequency of once per second. After that, the diameters of the sample in two perpendicular directions were measured with a caliper, and the arithmetic mean value was taken as the fluidity index of the sample under this water consumption. It was found in the test that the fluidity of the sample was roughly between 136 mm and 145 mm, with little fluctuation in the range, but none of them met the work ability index requirements specified in Table 2. In addition, during the mixing process, it was found that the mixture was too loose and difficult to be formed. Based on this analysis, the water-binder ratio in the initial mix proportion proposed in Table 5 was too low. The research made targeted adjustments based on this, and finally obtained a suitable water-binder ratio of 0.17, increased the dosage of the high-range water reducer from the original 0.5% to 1.0%, and the ratio of sand to binder was 1.2. On the premise of keeping the water-binder ratio unchanged, the dosages of other materials were slightly adjusted to obtain four groups of optimized mix proportions 1, 2, 3, and 4, as shown in Table 5.

The fluidity test was carried out on the optimized mix ratio, and the results of the fluidity test are shown in Table 6.

According to the workability indexes specified in Table 2, the fluidity test results of the four groups of optimized mix proportions all meet the design requirements of UHPC. When the fluidity is between 163 and 185 mm, it can be reasonably formed, and the formed specimens have a dense, uniform and complete appearance, without bleeding. Therefore, in the subsequent research, the four groups of optimized mix proportions obtained from the trial mixing are selected to further analyze the durability indexes of UHPC and the variation laws of its different influencing factors.

## 3. Study on the Durability Performance of UHPC

### 3.1. Test Materials

The test raw materials include P·O42.5 ordinary Portland cement, Class II fly ash produced in Jinan, silica fume, S95 grade slag powder produced in Jinan, standard sand, industrial quartz sand with coarse particle size (1.25–0.63 mm), industrial quartz sand with medium particle size (0.63–0.315 mm), polycarboxylic acid solid powdered water reducer produced in Shandong, and straight short steel fibers. Parameters are shown in Table 7, Table 8, Table 9, Table 10, Table 11, Table 12, Table 13, Table 14 and Table 15.

### 3.2. Test Methods and Procedures

#### 3.2.1. Resistance to Chloride Permeability

**Figure 3:**
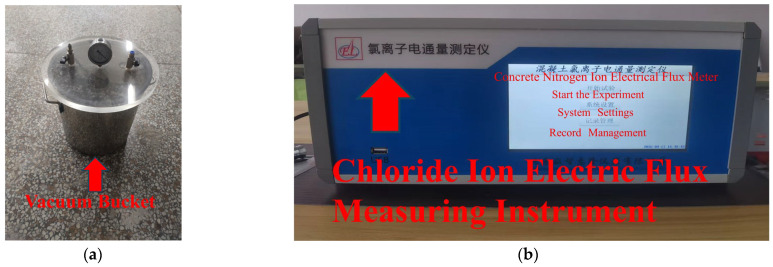
Impermeability test device. (**a**) Vacuum barrel, (**b**) Electric flux device.

The chloride ion permeability resistance of UHPC was evaluated in accordance with the Standard Test Method for Long-Term Performance and Durability of Ordinary Concrete (GB/T 50082-2009) [35], employing the electric flux method to determine the total charge passed through the concrete specimens. The primary testing apparatus is the chloride ion penetration resistance testing system, as illustrated in Figure 3. Specifically, Figure 3a shows the vacuum saturation chamber, while Figure 3b presents the electric flux measurement device.

**Figure 4:**
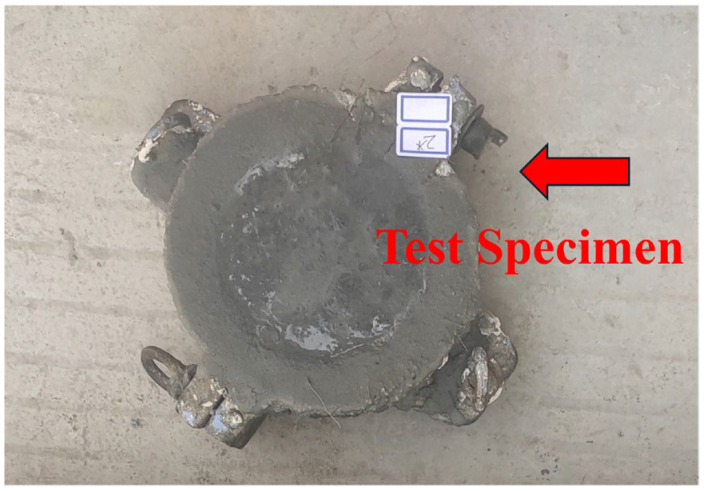
Marine concrete electric flux test block.

Prior to testing, any conductive materials on the specimen surface, such as exposed steel fibers, were carefully removed to avoid interference with electrical measurements. A total of 24 specimens were cast into cylindrical shapes with a diameter of 100 ± 1 mm and a height of 50 ± 2 mm. The prepared specimens are presented in Figure 4.

In the test, the specimen is first placed in a vacuum tank to complete the treatment and soaking process, and then it is loaded into the test cell. Subsequently, a 0.3 mol/L NaOH solution and a 3% NaCl solution are respectively poured into the positive and negative poles of the test cell. The voltage is adjusted to 60 ± 0.1 V, and the total current value passing through the specimen is recorded. Finally, by substituting the relevant data into Equation (2), the total electric flux value of the specimen is calculated.

(2) $$Q=\frac{900}{1000}(I_{0}+2I_{0}+2I_{90}+\cdot \cdot \cdot +2I_{270}+2I_{300}+I_{360})$$

where D is the total electric flux through the specimen (C); $I_{0}$ is initial current (A), accurate to 0.01 A; $I_{t}$ is Current (A) at time t (min), accurate to 0.01 A.

For the purpose of unified comparison, the calculated total electric flux passing through the specimens is transformed into the electric flux value of a sample having a diameter of 95 mm through Equation (3).

(3) $$Q_{95}=Q_{100}\times {\left(\frac{95}{100}\right)}^{2}$$

where $Q_{95}$ is the electric flux (C) through the specimen with a diameter of 95 mm; $Q_{100}$ is electric flux (C) through a specimen with a diameter of 100 (mm).

In accordance with the provisions of Reference [35], the total electric flux value is employed to evaluate the resistance to chloride ion penetration of different UHPC specimens. The smaller the total electric flux value of the specimen, the better the chloride ion penetration resistance of UHPC.

According to the experimental design, different specimens were first cured for 28 days through two methods, namely the standard curing method and the high-temperature curing method, and then the vacuum water saturation test and the electric flux test were carried out. The vacuum water saturation test was carried out using a vacuum barrel with a wall thickness of 1.4 mm, a bottom diameter of 300 mm, and a height of 300 mm. After the specimen was placed in the barrel, the vacuum degree was maintained for 3 h. While the equipment was running continuously, distilled water was added until the test piece was plunged into the liquid. Subsequent to 1 h, the normal pressure was restored, and the specimen was continuously soaked for 12 ± 2 h, which was regarded as the completion of water saturation. After the water saturation was completed, the test of electric flux was carried out, with a total duration of 6 h. The existing current magnitude was recorded every 30 min until 360 min. The arithmetic mean value of the three specimens in the same group was regarded as the ultimate measured value.

It was found in the test that the specimens exhibited a strength retraction phenomenon after being cured at high temperature for 28 days. Therefore, to avoid damage to the specimens caused by excessive curing time, the specimens cured for 28 days were selected for the performance tests in the durability tests of UHPC.

#### 3.2.2. Resistance to Sulfate Attack

The sulfate resistance of UHPC was evaluated in accordance with the Standard Test Method for Long-Term Performance and Durability of Ordinary Concrete (GB/T 50082-2009) [35], in order to assess its durability under sulfate exposure conditions, and A total of 48 cubic specimens with dimensions of 100 mm × 100 mm × 100 mm were prepared. Among them, 24 specimens were assigned to the control group for the determination of initial compressive strength. If any measured value deviated by more than 10% from the mean, it was considered an outlier and excluded. The mean was then recalculated based on the remaining values. If an outlier still existed among the remaining five values after exclusion, the entire set was regarded as invalid. Soaking the specimens in the Na_2_SO_4_ solution, air-drying and drying them are defined as one wet-dry cycle. The time of each cycle does not exceed 24 h, and the pH value of the Na_2_SO_4_ solution is maintained between 6 and 8. After the cycle is completed, the measured values are substituted into Formula (4) to calculate the corrosion resistance coefficients of the compressive strength of different specimens. The maximum number of dry-wet cycles when the corrosion-resisting coefficient regarding the compressive strength of the test piece is not less than 75% is used to determine the sulfate resistance grade of UHPC.

(4) $$K_{f}=\frac{f_{cn}}{f_{co}}\times 100\%$$

where $K_{f}$ is Corrosion resistance coefficient of compressive strength (%); $f_{cn}$ is the measured value of compressive strength (MPa) of a group of concrete specimens corroded by sulfate after N dry-wet cycles, which is accurate to 0.1 MPa; $f_{co}$ is the compressive strength of a group of concrete specimens compared with the standard curing of the same age of the sulfate-corroded specimens (MPa), accurate to 0.1 MPa.

**Figure 5:**
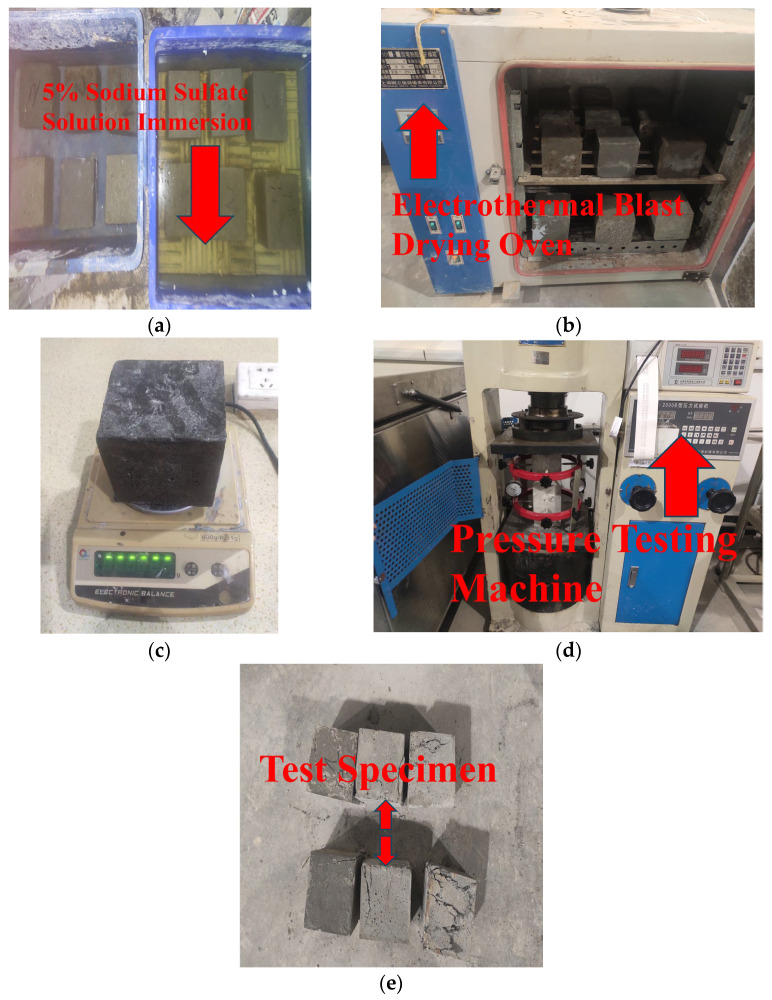
Test process of sulfate corrosion resistance. (**a**) Immersion in 5% sodium sulfate solution, (**b**) Oven low temperature drying, (**c**) Weighing, (**d**) Compression test, (**e**) Compressive failure of specimens.

Following the curing of the specimens under standard and high-temperature conditions for 28 days, 24 specimens among them were initially designated as the control group to measure the initial compressive strength. The final measured value was determined as the average value of the three specimens within the same group. Then, the remaining specimens are submerged in a 5% Na_2_SO_4_ solution and dried. A total of 120 dry-wet cycles are designed in the test. The specimens are weighed every 30 cycles, and their mass change rates are recorded. After the 120 dry-wet cycles are completed, the compressive strength of the specimens is determined. The main experimental procedure is shown in Figure 5.

#### 3.2.3. Frost Resistance

The frost resistance of UHPC was tested in accordance with the Standard Test Method for Long-Term Performance and Durability of Ordinary Concrete (GB/T 50082-2009) [35], to evaluate its durability under freeze–thaw cycling conditions. A total of 48 cubic specimens with dimensions of 100 mm × 100 mm × 400 mm were prepared. Among them, 24 specimens were assigned to the control group for the determination of initial compressive strength. If any measured value deviated by more than 10% from the mean, it was considered an outlier and excluded. The mean was then recalculated based on the remaining values. If an outlier still existed among the remaining five values after exclusion, the entire set was regarded as invalid. First, the specimens are cured for 24 days and then immersed in water. After 4 days, they are taken out for the determination of the initial mass. Then, the specimens are placed in a freeze-thaw test chamber for freeze-thaw cycles. Each freeze-thaw cycle is completed within 2 to 5 h. The freezing temperature range is −20 °C to −15 °C, and the thawing temperature range is 15 °C to 20 °C. Once the freeze-thaw cycles have been accomplished, the strength at which the specimens can withstand compression is measured. Finally, the rate of loss of compressive strength and mass loss rate of the specimens are calculated according to Formulas (5) and (6) respectively.

(5) $$\Delta f_{c}=\frac{f_{co}-f_{cn}}{f_{co}}\times 100\%$$

where $\Delta f_{c}$ is the compressive strength loss rate (%) of the specimen after *n* freeze-thaw cycles; $f_{co}$ is compressive strength (MPa) of specimens without freeze-thaw cycles; $f_{cn}$ is the compressive strength (MPa) of $f_{cn}$ -after n freeze-thaw cycles.

The mass change rate of the specimen is calculated as follows:

(6) $$W=\frac{m_{0}-m_{n}}{m_{0}}\times 100\%$$

where W is the mass change rate of the specimen after n freeze-thaw cycles (%); $m_{0}$ is the mass of specimen before test (kg); $m_{n}$ is the mass of the specimen after n freeze-thaw cycles (kg).

**Figure 6:**
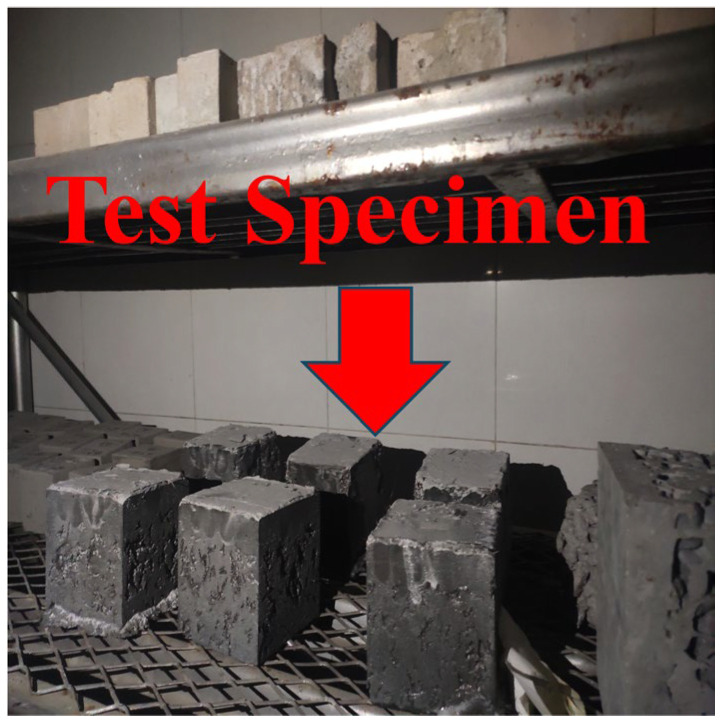
Marine concrete freeze-thaw cycle test block.

**Figure 7:**
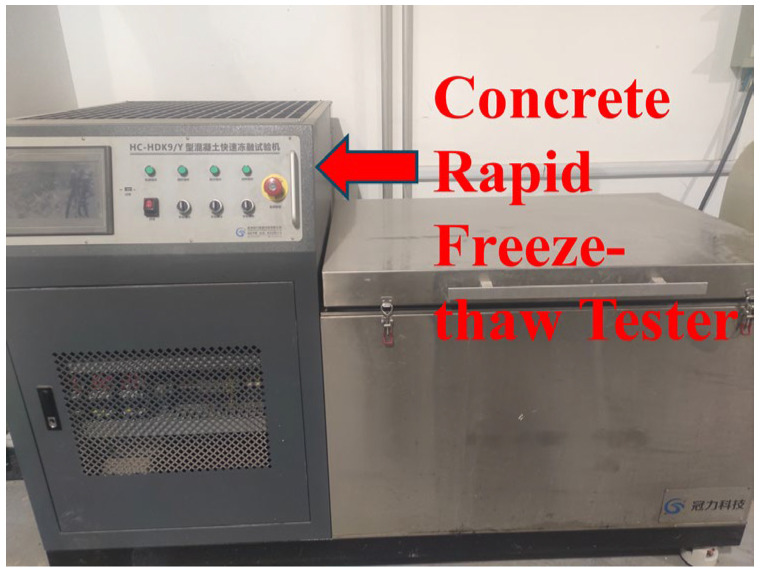
Freeze-thaw testing machine.

The test instrument used is the HC-HDK9/Y type rapid freeze-thaw testing machine for concrete (Hangzhou Guanli Intelligent Technology Co., Ltd. and Jianyuan Huace Technology (Hangzhou) Co., Ltd., Hangzhou, China). The test modules and the instrument are shown in Figure 6 and Figure 7. Once the specimens have undergone standard and high-temperature curing for 28 days, the initial compressive strength of the specimens is measured first. Subsequently, the freeze-thaw cycling process commences. The UHPC specimens are weighed every 100 cycles, and after 500 cycles are finished, the compressive strength of these specimens is ascertained.

#### 3.2.4. Electron Microscope Test

To further acquire the microscopic expressions of the durability properties of UHPC under various influencing factors, on the basis of the preceding physical experiments, this research employs a Scanning Electron Microscope (SEM) to perform internal scans on the specimens saved from relevant performance tests. From a microscopic standpoint, it analyzes the hydration products of UHPC with different configurations and observes the internal damage patterns, with the expectation of conducting a related analysis of the relationships between the hydration reaction mechanisms of UHPC under different factors, the variations in structural traits, and its durability. It serves as a fundamental reference for the selection of UHPC raw materials, the formulation of mix proportions, and its practical applications in engineering.

**Figure 8:**
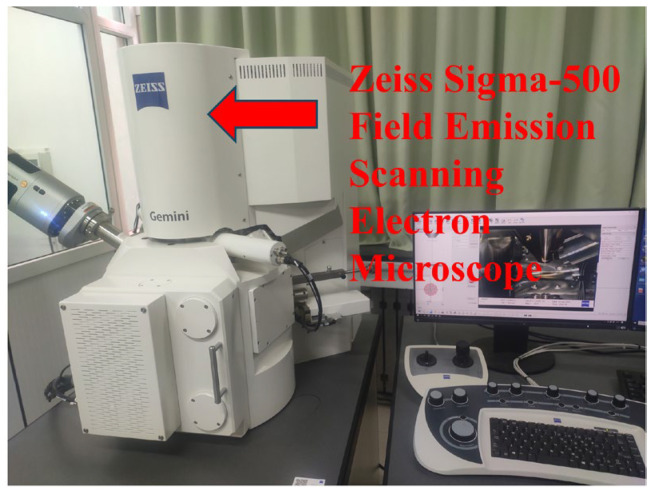
Electron microscope.

The electron microscope employed in this study’s experiment is the Zeiss Sigma-500 field emission scanning electron microscope (SEM, Carl Zeiss Microscopy Zeiss AG, Oberkochen, Germany). As depicted in Figure 8, the test specimen utilizes the raster scanning method. Initially, a focused high-energy electron beam is applied to hit the surface of the sample under test. All kinds of physical information are stimulated by the interaction between the beam and the material. Subsequently, the detector collects the secondary electrons emitted from the sample surface. Eventually, a two-dimensional image is shown and formed in accordance with the time sequence of the electron beam scanning, thereby enabling the internal observation of the microscopic structure of the hardened cementitious material of UHPC.

## 4. Analysis of Test Results

### 4.1. Analysis of the Results of the Resistance to Chloride Penetration Test

**Figure 9:**
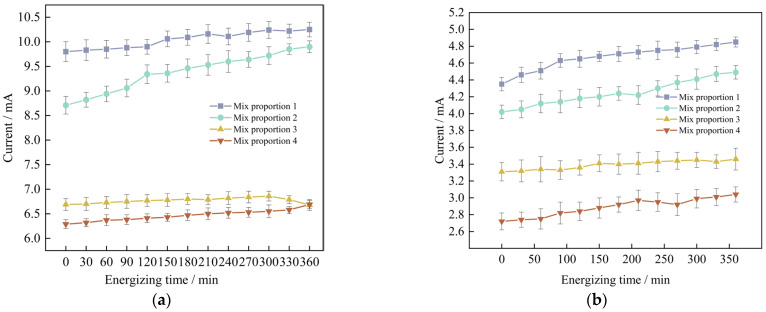
The measurement results of the current change with time of marine concrete at different curing temperatures are adopted. (**a**) Standard maintenance environment, (**b**) High temperature curing environment.

The results of the electric flux tests of four groups of UHPC in the situations of standard curing and high-temperature curing, the relevant data are demonstrated in Figure 9a and Figure 9b respectively. The data represent the values of the current of the specimens changing with time.

**Figure 10:**
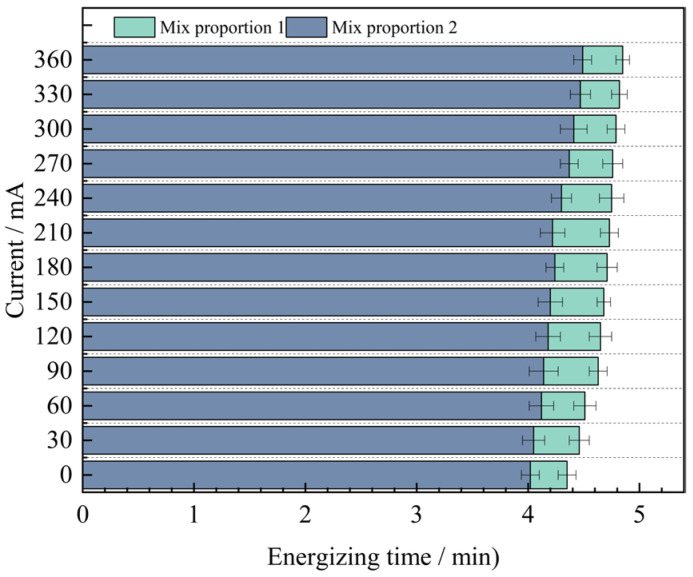
The variation value of concrete current with time under high temperature curing condition with different sands.

Through analysis, it is found that for the anti-chloride ion penetration performance of the specimens with four groups of mix proportions designed in the test, the current value at 360 min under the standard curing condition is 1.9 to 2.2 times the current value under the high-temperature curing condition. Evidently, the anti-chloride ion penetration property of the specimens under the high-temperature curing condition is much superior to that under the standard curing condition. Based on the high-temperature curing, the alterations of the current values of the mix proportions 1 and 2 with time are shown in Figure 10.

As can be seen from Figure 10, under the condition of high-temperature curing, the anti-chloride ion erosion performance of UHPC with quartz sand added is better than that of UHPC with standard sand added. The test results show that when industrial quartz sand is used to replace standard sand, the average value of the current passing through the concrete at different times is reduced by 0.42 mA. The difference between the two displays an inclination of increasing first and then decreasing. The difference is the largest at 210 min, reaching 0.51 mA. The reason for the analysis is that the quartz sand is composed of two kinds of graded particle sizes. When making the specimens, the fine sand can fully fill the pores between the coarse sand particles. Compared with the concrete specimens with standard sand added, it has stronger compactness, resulting in a smaller number of pores in the concrete. It effectively cuts off the routes for chloride ions to penetrate into the concrete, thus reducing the chloride ion penetration performance. Therefore, the resistance to chloride ion penetration performance of Mix Proportion 2 is better.

**Figure 11:**
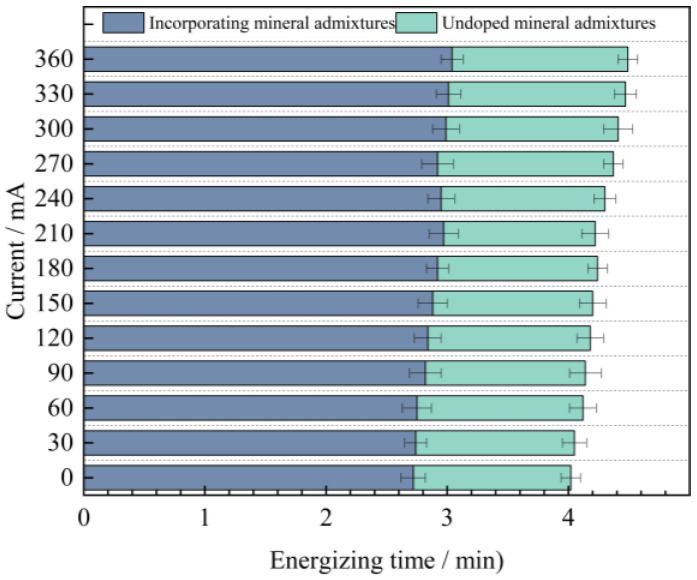
The change value of concrete current with time whether the admixture is added or not.

In order to further explore the influence of mineral admixtures on the anti-chloride ion erosion performance of UHPC, based on the previous research, the relationship between the current values of mix proportions 2 and 4 and time under the condition of high-temperature curing was obtained, as shown in Figure 11.

As can be seen from Figure 11, when industrial quartz sand is used as the fine aggregate and mineral admixtures are added to UHPC, the average value of the current passing through it decreases by 2.76 mA. Moreover, as time goes by, the difference between the two gradually increases, and it reaches the maximum value of 1.45 mA at 360 min. From this, it can be concluded that mineral admixtures are more helpful for the improvement of the anti-chloride ion penetration performance of UHPC in the later stage.

Through a comprehensive comparison of the four groups of mix proportions, it is evident that Mix Proportion 4, which utilizes quartz sand as the fine aggregate and incorporates mineral admixtures under high-temperature curing conditions, exhibits the lowest current value. At 360 min, the measured current is only 3.04 mA. This result indicates that the combined effect of adding mineral admixtures and applying high-temperature curing significantly enhances the compactness of the concrete, thereby improving the resistance of UHPC to chloride ion penetration.

**Figure 12:**
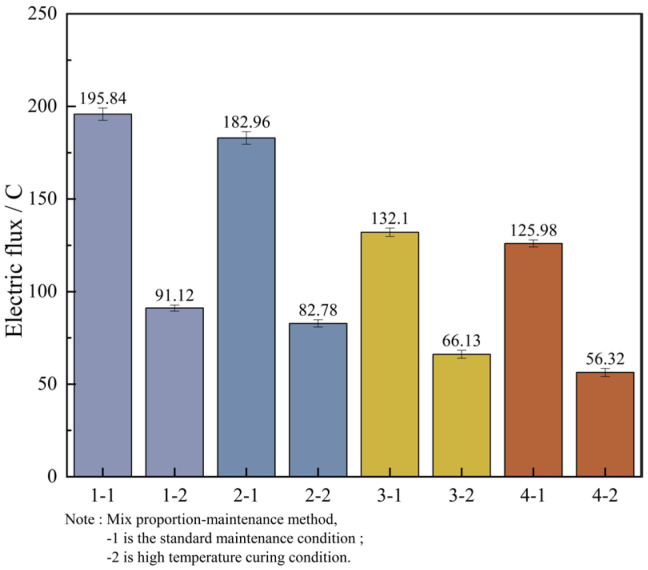
Measurement results of electric flux of concrete cured at different temperatures.

In order to facilitate a unified comparison, the total electric flux values obtained through calculations were normalized and converted to produce Table 16. Based on these converted values, bar charts of electric flux for each mix proportion were plotted, as illustrated in Figure 12.

By analyzing Figure 12, it can be known that the variation laws of the magnitudes of the electric flux of the four groups of mix proportions are roughly the same. In the high-temperature curing environment, the electric flux of UHPC is significantly reduced, especially for mix proportions 1 and 2. They are respectively reduced by 53.5% and 54.8% compared with the standard curing, but the difference in the electric flux values between the two is not large. Evidently, high-temperature curing can significantly enhance the resistance of UHPC to chloride ion erosion, while the variation in the type of fine aggregate has a minor influence on it. The main reason for this is that in a high-temperature environment, the hydration reaction speed of cement is increased, which contributes to the promotion of crystal growth within the hydration products, resulting in finer crystallization and the formation of more hydration products. As a result, the matrix structure becomes denser and more homogeneous, thereby reducing the internal voids and enhancing the anti-permeability ability. In addition, high-temperature curing can also promote the discharge of water inside the concrete and reduce the internal water content. Water is likewise one of the carriers for the penetration of chloride ions. Decreasing the water content in the concrete can lead to a reduction in the penetration rate of chloride ions.

In addition to the fact that the energy efficiency of high-temperature curing reduces the electric flux of UHPC, it is not difficult to see from the figure that the electric flux values of the last two groups of mix proportions are much smaller than those of the first two groups. Under the standard curing conditions, the electric flux values of mix proportions 3 and 4 decreased by 32.5% and 31.1% respectively. Under the high-temperature conditions, they decreased by 27.4% and 32.0% respectively. This is mainly because mineral admixtures were incorporated into the UHPC. Thus, mineral admixtures are capable of enhancing the resistance of UHPC to chloride ion penetration effectively. The improvement of UHPC by admixtures is mainly manifested in two aspects. One is that the addition of mineral admixtures makes the matrix structure of UHPC more refined, strengthening its capacity to fend off the intrusion of harmful ions. Secondly, mineral admixtures possess a certain degree of activity. Their components can serve as the nucleus of the hydration reaction in UHPC, enabling cement particles to more readily form the crystallization nuclei of hydration products on their surfaces. As a result, the cement hydration reaction is more swift and effective in the initial stage, which contributes to speeding up the whole hydration reaction process, generating more cementitious substances, enhancing the compactness and strength of UHPC, and thereby decreasing the permeability of chloride ions.

Table 17 presents the anti-chloride ion erosion grades of ordinary concrete in Reference [27].

Based on the aforementioned evaluation criteria, the chloride ion permeability of all four UHPC mix proportions falls within the “extremely low” category. Under standard curing conditions, the electric flux values of Mix Proportions 3 and 4 are both reduced to below 100 C. Under high-temperature curing, the electric flux values of all four mix proportions drop to below 100 C, with Mix Proportions 3 and 4 achieving values below 50 C. This indicates that their durability performance reaches the level where chloride ion permeability can be considered negligible according to the relevant standards.

### 4.2. Analysis of Sulfate Erosion Resistance Test Results

#### 4.2.1. Mass Loss from Dry and Wet Changes

**Figure 13:**
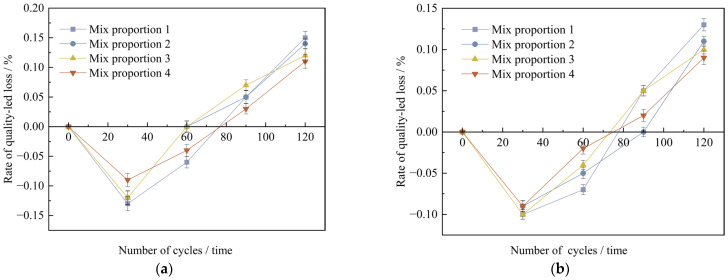
The mass change value of concrete specimens with different dry-wet cycles under non-curing conditions. (**a**) Standard curing conditions, (**b**) High temperature curing conditions.

The research shows that when the UHPC specimens are in an environment of sulfate erosion, as time goes by, the surface of the specimens will exhibit different degrees of peeling of cement paste and fine aggregates, resulting in a decrease in the mass of the specimens. The test obtained the mass change rates of UHPC specimens under different curing conditions after 30, 60, 90, and 120 wet-dry cycles, as shown in Figure 13a,b.

As can be seen from Figure 13, under the two conditions of standard curing and high-temperature curing, the mass change rates of UHPC specimens are generally the same. With the increase in the number of wet-dry cycles, the mass change of the specimens shows a trend of first increasing and then decreasing. Starting from the 90th wet-dry cycle, the specimens gradually experience mass loss. The analysis reveals that there are mainly two reasons for the initial increase in the mass of the specimens: Firstly, there may be tiny pores on the surface of the concrete specimens. When the specimens start to be immersed in the Na_2_SO_4_ solution, these pores may absorb water, which may lead to a slight increase in the mass of the specimens in the initial stage. Secondly, the SO_4_^2−^ in the sodium sulfate solution may undergo a chemical reaction with the cement hydration products or unhydrated cement clinker particles to form ettringite and gypsum. These compounds precipitate on the surface of the specimens in solid form in the initial stage of the test, thus causing a slight increase in the mass of the specimens. Later on, SO_4_^2−^ reacts with the calcium content on the surface of cement particles, which brings about alterations in the surface of the specimen particles and influences the bonding strength among the particles, leading to the peeling of the specimen surface. Moreover, the corrosion of the Na_2_SO_4_ solution can make cracks and cavities emerge on the surface of the specimen, consequently causing the mass loss of the specimen.

By comparing the mass loss rates of the specimens after 120 wet-dry cycles, it is evident that, under high-temperature curing, all four mix proportions exhibit reduced mass loss compared to standard curing, with reductions ranging from 13.3% to 21.4%. Specifically, when industrial quartz sand is used to replace standard sand, the mass loss rates under the two curing conditions decrease by 6.7% and 15.4%, respectively. Moreover, incorporating mineral admixtures into the concrete results in an average 20% reduction in mass loss due to wet-dry cycles. Based on the above analysis, high-temperature curing, fine aggregate substitution, and the addition of mineral materials can all improve the ability of concrete to resist sulfate erosion to a certain extent.

#### 4.2.2. Loss of Compressive Strength Due to Dry and Wet Changes

**Figure 14:**
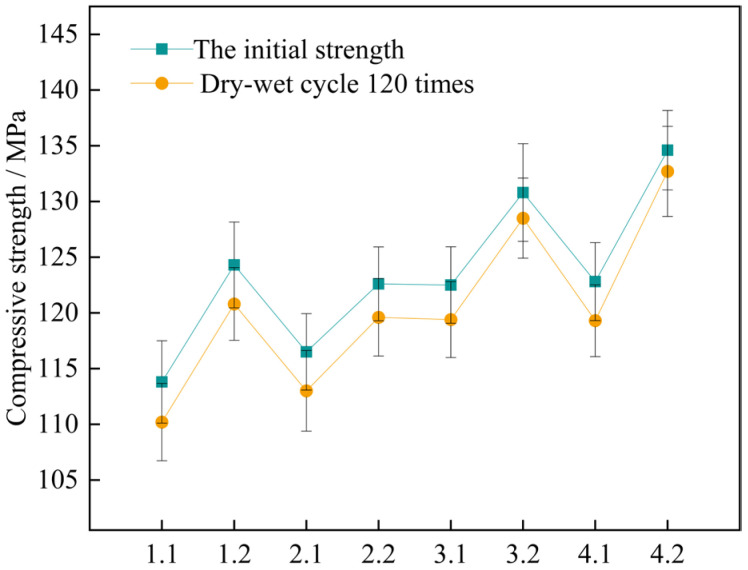
Changes in compressive strength of concrete specimens for 120 wet and dry cycles. Note: Mix proportion-maintenance method, -1 is the standard maintenance condition; -2 is high temperature curing condition.

Studies have shown that as the wet-dry cycles progress, while the mass of UHPC specimens decreases, their compressive strength also shows a certain degree of reduction. In order to describe in detail the change in the compressive strength of the specimens during the wet-dry change process, the research recorded the initial strength value of the concrete specimens and the compressive strength value after 120 cycles. Plot a diagram depicting the relationship between the wet-dry cycles and the loss of compressive strength, which is illustrated in Figure 14.

The findings indicate that for the two curing conditions, the four sets of mix proportions all comply with the corrosion resistance regulation in the standard. This standard requires that the lowest number of wet-dry cycles should exceed 120 times. When under the high-temperature curing condition, the loss values of the compressive strength of the four groups of mix proportions numbered 1, 2, 3, and 4 have dropped by 2.7%, 14.3%, 25.8%, and 45.7% respectively, as compared with the standard curing. It shows that high-temperature curing can help enhance the compressive property of concrete. By making a comparison between mix proportion 1 with standard sand added and mix proportion 2 with industrial quartz sand added, under the two curing conditions, the loss values of the compressive strength of the concrete vary within the range of 3.0 MPa to 3.6 MPa, showing little disparity. Evidently, the variation in the kind of fine aggregate has a negligible influence on the loss value of the concrete due to wet-dry cycles. For the two sets of mix proportions in which mineral admixtures are incorporated based on mix proportions 1 and 2, the loss values of the compressive strength have dropped. When under the high-temperature curing condition, following the wet-dry cycles, the compressive strengths of mix proportions 3 and 4 decrease by merely 2.3 MPa and 1.9 MPa respectively.

**Figure 15:**
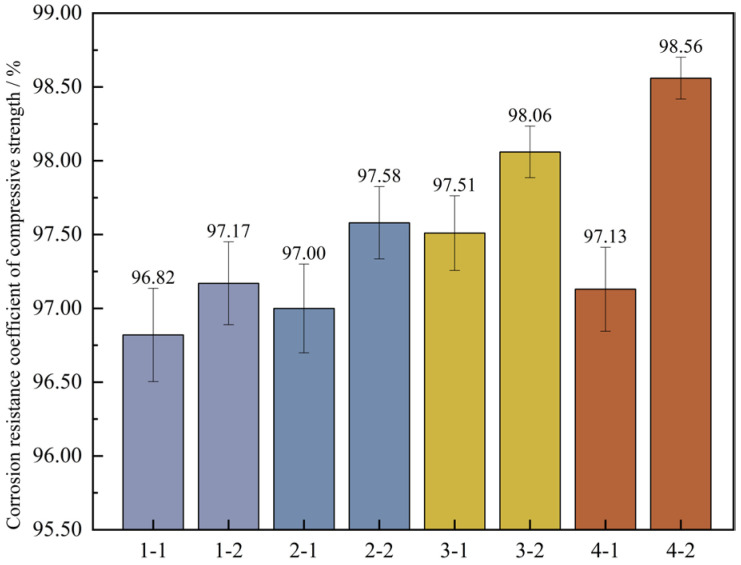
Corrosion resistance coefficient of concrete compressive strength. Note: Mix proportion-maintenance method, -1 is the standard maintenance condition; -2 is high temperature curing condition.

To further ascertain the changing pattern of the compressive strength of UHPC after undergoing wet-dry cycles, the corrosion resistance coefficient of the concrete’s compressive strength was computed based on Formula (4). Then, a bar graph depicting the wet-dry cycles and the corrosion resistance coefficient of the compressive strength was plotted, which is illustrated in Figure 15.

Figure 15 shows that under the high-temperature curing environment, the corrosion resistance coefficients of the compressive strength of the four groups of mix proportions have all increased to varying degrees, with an average growth rate of up to 0.73%. Among them, the corrosion resistance coefficient of mix proportion 4 has increased by 1.43%, which has the largest increase range. In the standard curing conditions, the corrosion resistance coefficient of the compressive strength of mix proportion 2 grew by 0.18% relative to that of mix proportion 1. When it comes to the high-temperature curing conditions, the difference between the two is 0.41%. It can be seen that replacing the standard sand with quartz sand has improved the corrosion resistance of the concrete. When compared with the first two groups of mix proportions, for mix proportions 3 and 4 in which mineral admixtures are incorporated, under the standard curing condition, the corrosion resistance coefficients of the compressive strength are raised by 0.69% and 0.13% respectively. Under the high-temperature curing condition, these coefficients are increased by 0.89% and 0.98% respectively. The incorporation of mineral admixtures has enhanced the sulfate erosion resistance of the concrete. Under the high-temperature curing conditions, the improvement effect is more significant, and the improvement effect is better than changing the type of sand.

The main reason is that the coarse aggregates have been removed from UHPC, and it only contains fine aggregates. The matrix is very dense, and there is no large interfacial transition zone. The meticulous particle distribution, the composition of particle sizes, together with the high-strength bonding materials, render it arduous for sulfates to seep into the inner part of the concrete. This compact structure lessens the pathways for sulfate corrosion, thereby enhancing the resistance to corrosion. Adding a substantial amount of mineral admixtures into UHPC is capable of optimizing the pore structure. The highly active silica fume reacts with the Ca(OH)_2_ produced during cement hydration to generate C-S-H gel, which ameliorates the interfacial performance between the fine aggregates and the matrix. When under high-temperature curing, it can further boost the reaction of cement hydration and silica fume. Meanwhile, the high temperature prompts some of the C-S-H gel to convert into tobermorite crystals with even more outstanding durability. Hence, UHPC cured at high temperature shows more excellent durability properties compared to that under standard curing conditions.

Based on a comprehensive analysis, it is revealed that under both standard curing and high-temperature curing conditions, the corrosion resistance properties of the four groups of mix proportions are able to satisfy the lowest requirement for resisting sulfates. Among them, mix proportion 4 under the high-temperature curing environment has the best performance, and mix proportion 3 ranks second. After being cured at high temperature, the corrosion resistance capabilities of mix proportions 1 and 2 can be approximately equal to those of mix proportions 3 and 4 under the standard curing conditions.

### 4.3. Analysis of Freezing Resistance Test Results

#### 4.3.1. Freeze-Thaw Cycle Mass Loss

The research used the approach of direct freezing-thawing to measure the frost resistance property of the specimens. When the mass loss rate of the specimen exceeds 5% or the compressive strength loss rate exceeds 25%, the freeze-thaw resistance performance of the specimen is determined to be unqualified. The test should be terminated in a timely manner. By calculating in accordance with formula (6), Table 18 is derived. It presents the mass variation rates of UHPC specimens that have undergone 100, 200, 300, 400, and 500 freeze-thaw cycles respectively, under various curing environments but the identical test conditions.

**Figure 16:**
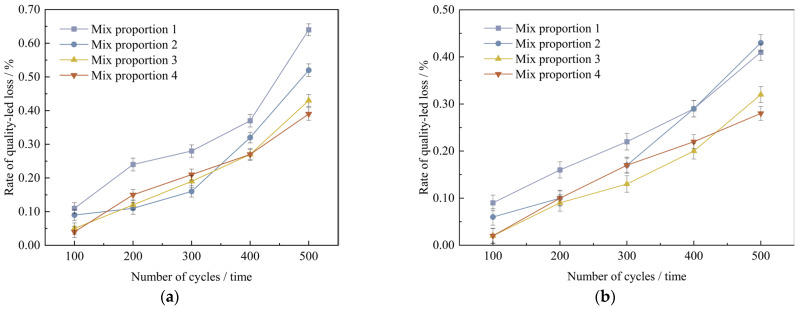
Quality change value of concrete specimens with different freeze-thaw cycles under curing conditions. (**a**) Standard curing conditions, (**b**) High temperature curing conditions.

Plot the curves depicting the variation relationship between the mass loss rate of UHPC and the times of freeze-thaw cycles, which are illustrated in Figure 16a,b.

As shown in Figure 16, under the two curing conditions, with the increase in the number of freeze-thaw cycles, the mass loss rate of UHPC gradually increases, but the mass loss rates of the four groups of mix proportions are all below 1%. In the standard curing conditions, mix proportion 1 has the highest mass loss rate. The reason is that the standard sand particles incorporated in mix proportion 1 are rather coarse and there are no mineral admixtures added, which leads to inferior compactness. After undergoing 500 freeze-thaw cycles, its mass loss is a bit higher than those of other mix proportions. Compared with the two mix proportions without mineral admixtures, the mass loss rates of mix proportions 3 and 4 with a large amount of mineral admixtures added are reduced by 0.21% and 0.25% respectively under the standard curing conditions, and reduced by 0.08% and 0.15% respectively under the high-temperature curing conditions. It can be concluded from this that mineral admixtures are capable of decreasing the mass loss of concrete to some degree. In the high-temperature curing conditions, following 500 freeze-thaw cycles, the mass loss rates of the four mix proportions decrease by 0.23%, 0.09%, 0.11%, and 0.11% respectively in comparison with those in the standard curing conditions. It indicates that high-temperature curing has a certain improvement effect on the frost resistance performance of UHPC.

#### 4.3.2. Loss of Compressive Strength in Freeze-Thaw Cycles

**Figure 17:**
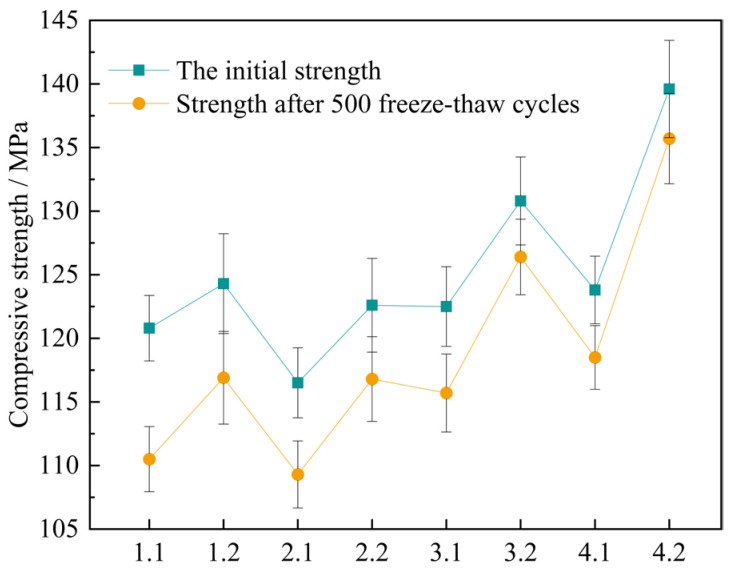
The change value of compressive strength of concrete specimens after 500 freeze-thaw cycles.

**Figure 18:**
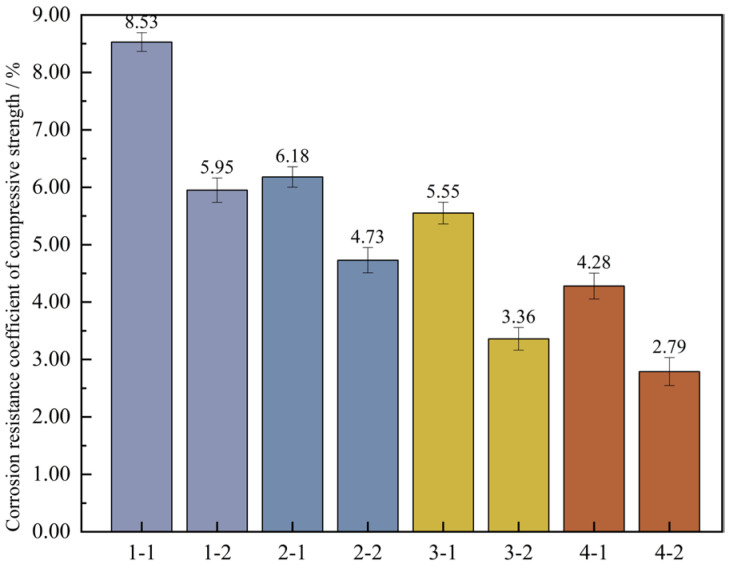
Loss rate of compressive strength of concrete. Note: Mix proportion-maintenance method, -1 is the standard maintenance condition; -2 is high temperature curing condition.

As the number of freeze-thaw cycles increases, the compressive strength of UHPC specimens gradually decreases. Table 19 shows the initial strength of the concrete specimens and the strength after freeze-thaw cycles. The loss rate of the compressive strength of UHPC specimens was obtained according to Formula (5). Plot the curve depicting the relationship between the number of freeze-thaw cycles and the loss amount of the compressive strength, along with the bar chart of the compressive strength loss rate, as illustrated in Figure 17 and Figure 18.

As indicated in Figure 17, after the four sets of mix proportions have experienced 500 freeze-thaw cycles, their compressive strengths have all declined. Nevertheless, in contrast to the standard curing, the degrees of decrease in the compressive strengths of several sets of mix proportions under the high-temperature curing condition are smaller. This demonstrates that high-temperature curing helps to improve the frost resistance property of UHPC. After 500 freeze-thaw cycles, the compressive strength of mix proportion 1 decreases by 10.3 MPa and 7.4 MPa respectively under the standard and high-temperature curing environments. The compressive strength of mix proportion 2 decreases by 7.2 MPa and 5.8 MPa respectively. It is not difficult to find that, regardless of the curing environment, the freeze-thaw resistance of mix proportion 2 is better than that of mix proportion 1. It can be seen that the incorporation of quartz sand contributes to the development of the frost resistance performance of UHPC. The main reason for this is that the particles of quartz sand are finer, resulting in a lower porosity of the fabricated concrete. It decreases the likelihood of the pores in the concrete absorbing water and expanding, and also reduces the probability of the increase in internal stress due to water infiltration, thereby enhancing the frost resistance property. Meanwhile, as can be observed from the figure, the compressive strength loss of mix proportions 3 and 4 is minimal, and the curves depicting the compressive strength after freeze-thaw and the initial strength of these two mix proportions nearly overlap. It has been demonstrated that mineral admixtures are also capable of effectively enhancing the frost resistance property of UHPC.

According to the Formula (6), the compressive strength loss rate of concrete specimens is calculated, and the histogram 17 is drawn.

It is known from Figure 18 that after high-temperature curing, the loss rates of compressive strength of each group of mixtures are 5.95%, 4.73%, 3.36%, and 2.79% respectively, with a distinct downward trend. The main reason for this is that high-temperature curing can speed up the hydration reaction process in UHPC, allowing cement particles to react with water at a faster pace to generate hydration products, and enhancing the density of UHPC within a short period, thereby decreasing the likelihood of micro-cracks emerging during freeze-thaw cycles. Moreover, high-temperature curing can also reduce the early shrinkage rate of UHPC. The early shrinkage of concrete may cause the internal stress in the concrete to concentrate, increasing the risk of crack formation.

Furthermore, it can be easily observed from the graph that, under the two kinds of curing conditions, the loss rates of compressive strength of the four sets of proportions decline in a linear manner, which shows that the frost resistance properties of these several sets of proportions are gradually enhanced. In the standard curing conditions, the loss rate of the compressive strength of mix proportion 2 decreases by 2.35% relative to that of mix proportion 1. In the high-temperature environment, it drops by 1.22%. Evidently, whether under the standard curing or the high-temperature curing, substituting the standard sand with quartz sand can effectively enhance the freeze-thaw resistance ability of the concrete. In the standard curing conditions, the loss rate of the compressive strength of mix proportion 3 incorporating mineral admixtures is 5.55%, and in the high-temperature environment, it is 3.36%. When compared with mix proportion 1, it decreases by 2.98% and 2.59% separately. As for mix proportion 4 which also has admixtures incorporated, it drops by 1.9% and 1.94% separately in comparison with mix proportion 2 in the two curing environments. From this, it can be concluded that the incorporation of mineral admixtures into UHPC is beneficial to enhancing its frost resistance. The reason lies in that the addition of mineral admixtures can alter the microstructure and mechanical characteristics of the concrete through means such as improving the pore structure, producing hydration products, and minimizing thermal cracks, and thus reducing the harm inflicted on the concrete by freeze-thaw cycles. The tiny particles of the mineral admixtures and the stable products formed during the hydration reaction process can fill certain pores within the concrete, enhancing the density of the concrete. Consequently, it decreases the likelihood of water seeping into the concrete’s interior. Reduce the formation of cracks caused by the expansion of water during freezing, thus improving the frost resistance of UHPC. In addition, the mineral admixtures can change the hydration process of UHPC and reduce the heat release rate of the concrete, that is, slow down the rate of temperature rise. Through decelerating the rate of the hydration reaction, heat production can be efficiently lowered, thereby lessening thermal stress and reducing the likelihood of thermal cracks. This is also helpful for the freeze-thaw resistance performance of the concrete, This is also helpful for the freeze-thaw resistance performance of the concrete. Thus, the incorporation of mineral admixtures is able to enhance the frost resistance performance of UHPC by means of multiple channels.

The research results show that the frost resistance performances of the four groups of mixtures prepared in the experiment can all meet the requirements. The mix proportion 4 under high-temperature curing has the best performance, and mix proportion 3 comes second. In addition, the freeze-thaw resistance performance of mix proportions 1 and 2 is improved after high-temperature curing, and there is not much difference between them and mix proportions 3 and 4 under standard curing. However, under the standard curing environment, the frost resistance performances of Mixture Proportion 1 and Mixture Proportion 2 are poor, and they should be used with caution.

### 4.4. Research on the Representation of Microscopic Performances of UHPC

**Figure 19:**
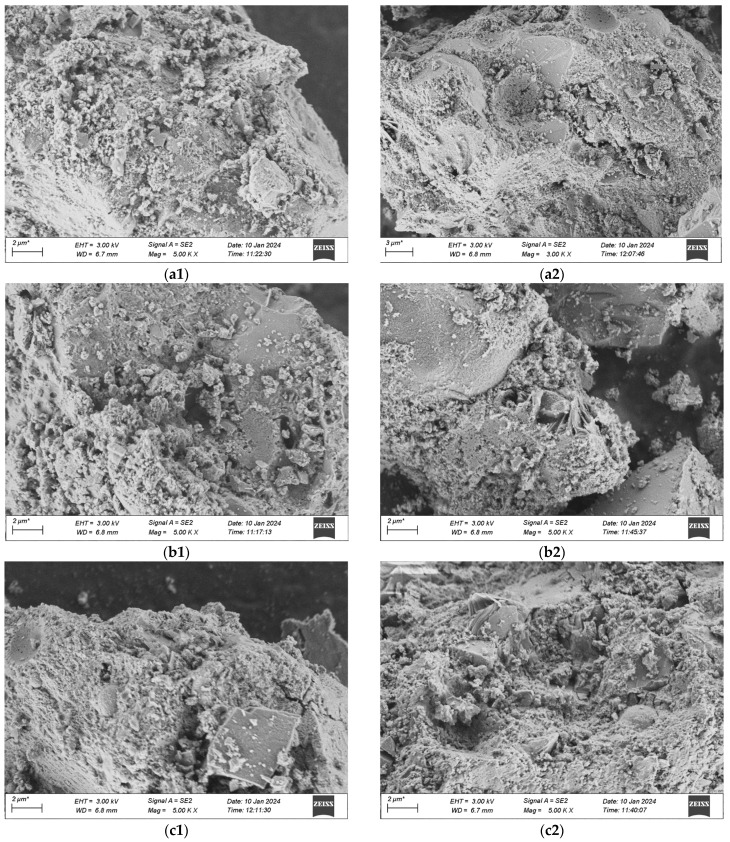

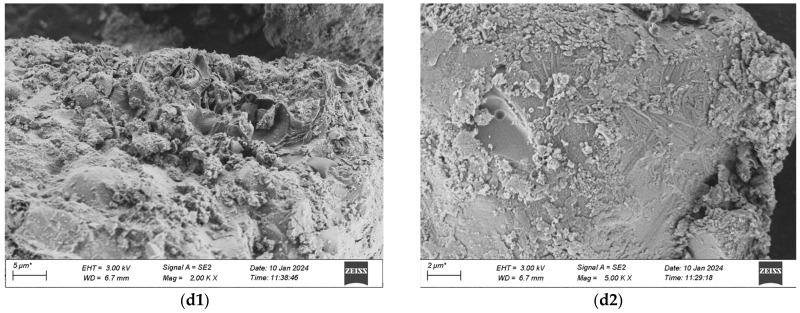
SEM Pictures of UHPC Materials in Various Combinations at the Age of 28 Days. (**a1**) 1-1 Group standard maintenance, (**a2**) 1-2 Combined with high temperature curing, (**b1**) 2-1 Group standard maintenance, (**b2**) 2-2 Combined with high temperature curing, (**c1**) 3-1 Group standard maintenance, (**c2**) 3-2 Combined with high temperature curing, (**d1**) 4-1 Group standard maintenance, (**d2**) 4-2 Combined with high temperature curing.

The voltage for this scanning is uniformly 3 kV, and the working distance measures 6.7 mm. The scanning outcomes are presented in Figure 19a–d, where 1 stands for standard curing and 2 stands for high-temperature curing.

The microscopic pictures reveal that among the specimens of various mix proportions under the standard curing condition, numerous particles distributed disorderly are present in the matrix, which shows that the compactness of what is formed by the colloid and the aggregate is not enough. Moreover, in the specimens cured under standard conditions, there are still some hexagonal flaky CH crystals that have not completely taken part in the secondary hydration. This is mainly due to the fact that the water-binder ratio of UHPC is extremely low. When the water-binder ratio of the cement mortar is less than 0.44, the cement fails to be fully hydrated, and with the decrease of the water-binder ratio, the extent of cement hydration will gradually decline. In contrast, in a high-temperature environment, the speed of hydration increases. CH can thoroughly take part in the secondary hydration, producing a great deal of C-S-H gel. The quantity of dispersed cement particles reduces, and needle-like crystal ettringite (AFt) that is insoluble in water is generated on the surface of the cement particles, which is tightly linked to the matrix. When compared with the appearance of the specimens in the standard group, the pore area of the specimens in the high-temperature group keeps decreasing.

When comparing Figure 19a with Figure 19b, it is discovered that when preparing UHPC by substituting industrial quartz sand for standard sand, the compositions and microscopic appearances of the two are alike. Both are gel-like materials with changeable compositions and relatively poor crystallization states, and their degrees of compactness are relatively low. In contrast, more crystals of hydration products are generated in the No. 2 mixture under the high-temperature curing condition. This is mainly due to the fact that the industrial secondary quartz sand offers a greater surface area, and the cement is not enough to cover its reaction zone. Thus, the hydrate crystallization appearance of the No. 2 mixture shows a slightly larger size compared to that of the No. 1 standard sand concrete mixture, and there exists a situation where the remaining sand grains are enclosed. When compared with Figure 19a and Figure 19b, in Figure 19c and Figure 19d, it can be observed that in the No. 3 and No. 4 mixtures with the addition of mineral admixtures, an evident flocculent structure of C-S-H gel is generated, which shows that the hydration products of the mineral admixtures are alike to those of Portland cement. The distinction is that the contents of oxides like Ca, Si, Al, and Fe in the gel are different, and thus the hydration products with diverse crystal shapes are also not the same. Moreover, ettringite continues to hydrate, predominantly yielding flaky crystalline hydrate calcium aluminate (CAH), and a minor portion will be converted into square flaky monosulfate hydrate calcium sulfoaluminate (AFm). This reaction proceeds quickly and creates a loose reticular structure, which facilitates the rapid setting of the cement paste. When comparing the microscopic characteristics of the first two groups of mixtures, it is clearly evident that the cement stone matrix of UHPC with the addition of mineral admixtures is more compact. This is due to the fact that the finer particles of the mineral admixtures can form a more compact filling structure with the cement, and more hydration products can be produced during the secondary reaction with the cement colloid, thereby filling the voids in the matrix more densely. Similarly, it is observable that the structure of UHPC cured at a high temperature has a greater density, and the ranking of the quality of the structures of the four groups of mix proportions is 4-2 > 3-2 > 1-2 > 2-2.

**Figure 20:**
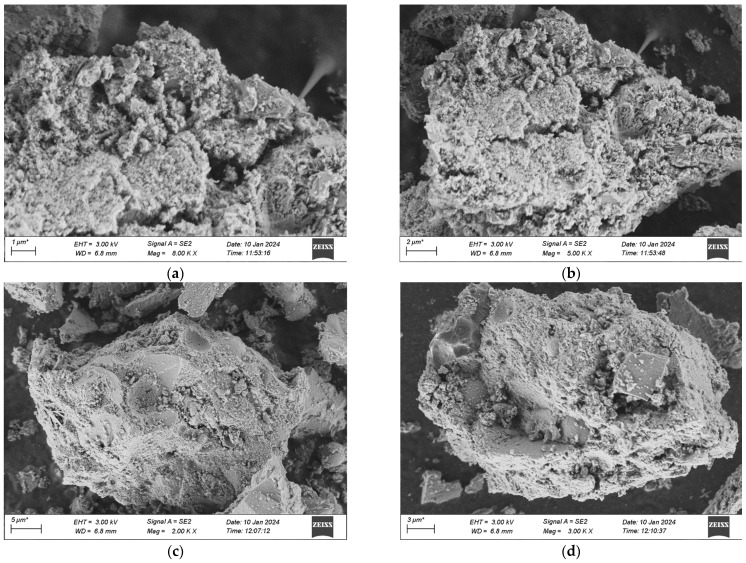
SEM morphology of each group after 500 freeze-thaw cycles. (**a**) 1 Interfix, (**b**) 2 Interfix, (**c**) 3 Interfix, (**d**) 4 Interfix.

**Figure 21:**
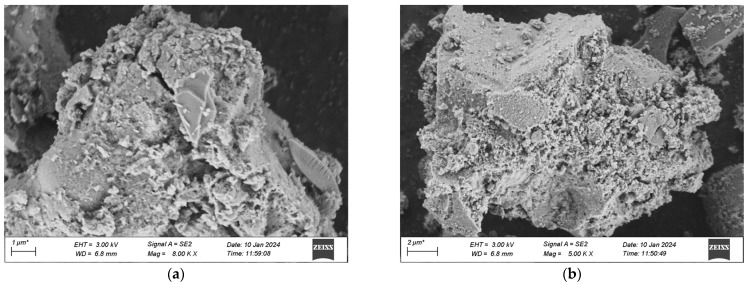

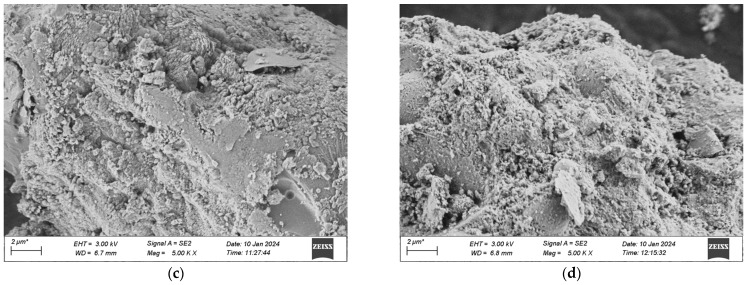
SEM morphology of each group after 120 dry-wet cycles. (**a**) 1 Interfix, (**b**) 2 Interfix, (**c**) 3 Interfix, (**d**) 4 Interfix.

**Figure 22:**
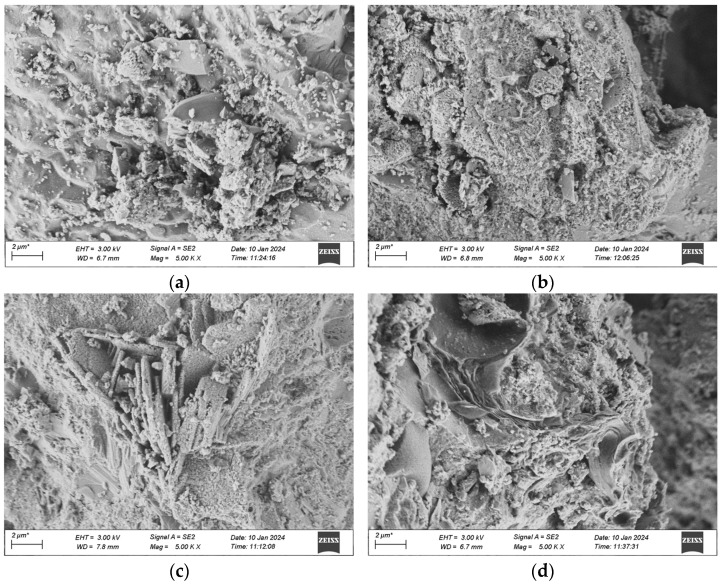
SEM morphology of samples after anti-permeability test of each group. (**a**) 1 Interfix, (**b**) 2 Interfix, (**c**) 3 Interfix, (**d**) 4 Interfix.

The samples for the microscopic analysis of the durability of UHPC regarding frost-thaw resistance, erosion resistance, and impermeability performance are predominantly sourced from the test specimens that have been cured in a high-temperature environment for 28 days. The contrast of the microscopic appearances of the UHPC specimens in each group before and after the experiment is presented in Figure 20, Figure 21 and Figure 22.

Figure 20 indicates that after 500 freeze-thaw cycles, the microscopic structures of the four groups of specimens have undergone changes in different degrees. Among them, it is observable that the specimens of Mix Ratio 1 and Mix Ratio 2 both exhibit distinct transverse cracks, and the right sides of the specimens of Mix Ratio 3 and Mix Ratio 4 show slender cracks, demonstrating that under the effect of freeze-thaw damage, the pores crack initially and micro-cracks are formed. The presence of the cracks further facilitates the infiltration of the solution, finally resulting in the destruction of the dense matrix structure within the UHPC. This is the primary cause of the reduction in the strength of UHPC. In the later phase of the freeze-thaw cycle, the formation of erosion products will generate expansion pressure, eventually making the specimens ineffective. By comparison, the quantity of dispersed cement particles in the UHPC of the No. 3 and No. 4 mixtures reduces, and the cracks are relatively small, which shows that the mineral admixtures can thoroughly take part in the hydration reaction in a high-temperature environment, and the tiny particles serve as a filling function. Evidently, although the addition of mineral admixtures might have an impact on the initial strength of UHPC, it is advantageous for the long-term improvement of its frost-thaw resistance.

As illustrated in Figure 21, after the dry-wet cycles, among the four groups of mixtures, obvious cracks were not observed in the groups other than Group 1. When examining the cracks, the generation of ettringite was detected, and there are two potential reasons: one is that the hydrated calcium aluminate produced by the hydration of cement reacts with gypsum, resulting in the formation of the original ettringite; the other is that during the dry-wet cycles, the specimen is immersed in the Na_2_SO_4_ solution, and SO_4_^2−^ seeps into the matrix and reacts with the hydration products of the cement to form secondary ettringite. Besides, the quantity of flaky CH crystals within the matrix has reduced, as SO_4_^2−^ in the solution will react with the CH crystals, thereby consuming a great deal of CH crystals.

Comparatively speaking, the gel structure in the UHPC of Mixture Proportion 2 is more compact and dense than that of Mixture Proportion 1, so no cracks are generated. It indicates that substituting quartz sand for standard sand in the preparation of UHPC can enhance the compactness and boost the ability to resist sulfate erosion. Moreover, the morphological alterations of UHPC after the dry-wet cycles are analogous to those after the freeze-thaw cycles. The structures of the two groups of mix proportions with the addition of mineral admixtures are more compact and the extent of damage is less severe than those without the addition of mineral admixtures.

As depicted in Figure 22, after the impermeability experiment, no distinct cracks were found in the matrix of the UHPC specimens in each group. Microscopic representation reveals that only pores with small sizes exist, and the structure is rather compact, which demonstrates that the specimens possess excellent impermeability properties, in accordance with the research findings on the chloride ion erosion resistance performance obtained in the previous experiments. Meanwhile, it can be seen that regular needle-like and rod-like crystals (AFt) are formed in each group of UHPC, and there are also numerous growing AFt, exhibiting a tendency of radial growth in all directions. Besides, a small quantity of irregular flakes are observed around the ettringite. It is speculated through analysis that they might be a small amount of irregular hexagonal flaky chloroaluminate produced by the infiltration of chloride ions. The existence of flaky crystals usually indicates that this part is relatively loose and has pores. As time goes on, this part may combine with the cross-net part to form holes.

## 5. Conclusions

(1) High-temperature curing and incorporation of mineral admixtures can efficiently enhance the chloride ion penetration resistance performance of UHPC. The 360-min current value of UHPC under high-temperature curing conditions is 1.9 to 2.2 times lower compared to that under standard curing conditions. When mineral admixtures are mixed into UHPC, the electric flux value decreases by about 30%. The kind of aggregate has a minor impact on the impermeability of concrete. The electric flux values of the concrete using industrial quartz sand and the concrete using standard sand are more or less identical.

(2) No matter what the curing conditions are, the change in the mass of UHPC follows a pattern of increasing initially and then decreasing. From the 90th dry-wet cycle onwards, the mass of the specimen starts to suffer a loss. After the specimen undergoes high-temperature curing, the average value of the compressive strength loss is decreased by 0.7 MPa. Substituting quartz sand for standard sand or adding admixtures can enhance the sulfate erosion resistance of concrete, yet the improvement effect of adding mineral admixtures in combination is superior.

(3) After 500 freeze-thaw cycles, the mass loss ratios of UHPC in the four groups of mix proportions are all kept below 1%. The frost resistance performance of UHPC is associated with the curing conditions, the kind of sand, and the mineral admixtures. Under high-temperature curing conditions, the average decrease rate of the compressive strength loss of UHPC after freeze-thaw cycles is 1.93%. When mineral admixtures are added, the average decrease rate of the compressive strength loss is 2.36%. When quartz sand is substituted for standard sand in concrete, the average decrease rate of the compressive strength loss is 1.79%.

(4) The research on microscopic characteristics reveals that the main hydration products of UHPC are C-S-H gel and calcium hydroxide. High-temperature curing can efficiently reduce the dispersed cement particles in the matrix and enhance the compactness of the matrix. Substituting industrial quartz sand for standard sand will result in a somewhat poorer condition of the hydration product crystals and pores of UHPC. Mineral admixtures deplete the content of calcium hydroxide through the hydration reaction, and their hydration products also fill the pores within the matrix. Under high-temperature curing conditions, the ranking of the quality of the microscopic appearance structures of UHPC in the four groups of mix proportions is 4 > 3 > 1 > 2. Each group of mix proportions exhibits excellent resistance to chloride ion penetration, and no distinct cracks are found in the SEM images. After 120 dry-wet cycles, apart from the occurrence of cracks in Mix Ratio 1, the microscopic appearances of the other mix ratios have not experienced remarkable changes. After the specimens have gone through 500 freeze-thaw cycles, the microscopic structures have all been impaired. Generally speaking, the structure of Mix Ratio 4 is damaged the least, has the optimal durability, and is more appropriate for application in marine engineering settings.

## Figures and Tables

**Table 1:** Design of the Dosage of Each Component of UHPC.

Grade	Water-Binder Ratio	Cementitious Material Content (kg/m^3^)	Steel Fiber Volume Fraction (%)	Silica Fume (g)	Cement (g)
UHPC	≤0.22	≤850	≥0.7	≥cementitious material content 10%	≥cementitious material content 50%

**Table 2:** Workability and mechanical performance indicators of UHPC.

Grade	Degree of Fluidity (mm)	Break Off Strength (MPa)	Compressive Strength (MPa)	Elastic Modulus (GPa)	Tensile Strength
UHPC	≥160	≥12	≥100	≥40	≥5

**Table 3:** Durability performance indicators of UHPC.

Freezing Resistance (Quick Freezing Method)	Resistance to Chloride Ion Permeability (C)	Sulfate Resistance
≥F500	Q ≤ 100	≥KS120

**Table 4:** Quartz sand bulk density and porosity.

Maximum Particle Size	Single Grain Grading			Continuous Grading		
Dmax (mm)	Apparent Density (kg/m^3^)	Bulk Density (kg/m^3^)	Porosity (%)	Apparent Density (kg/m^3^)	Bulk Density (kg/m^3^)	Porosity (%)
0.315	2659.30	1443.94	45.70	-	-	-
0.63	2667.37	1482.09	44.44	1540.67	1540.67	42.09
1.25	2646.35	1538.72	41.86	2651.28	1600.22	39.64

**Table 5:** Mix ratio.

Material Peer Group	W/B	Cement (g)	Coal Fly Ash (g)	Slag Powder (g)	Silica Fume (g)	Standard Sand (g)	Quartz Sand (Coarse) (g)	Quartz Sand (Middle) (g)	Steel Fiber (g)	Water Reducing Admixture (g)
Initial mix ratio 1	0.13	985	-	-	-	1170	-	-	180	4.93
Initial mix ratio 2	0.13	900	-	-	-	-	740	360	180	4.50
Initial mix ratio 3	0.13	576	189	63	72	1170	-	-	180	4.50
Initial mix ratio 4	0.13	630	202	68	90	-	791	339	180	4.95
Optimized mix ratio 1	0.17	985	-	-	-	1170	-	-	180	9.85
Optimized mix ratio 2	0.17	915	-	-	-	-	660	420	167	9.15
Optimized mix ratio 3	0.17	576	189	63	72	1070	-	-	164.5	9.00
Optimized mix ratio 4	0.17	630	202	68	90	-	810	358	180	9.90

**Table 6:** Mix ratio fluidity (mm).

Mix Proportion	Initial Mix Ratio 1	Initial Mix Ratio 2	Initial Mix Ratio 3	Initial Mix Ratio 4	Optimized Mix Ratio 1	Optimized Mix Ratio 2	Optimized Mix Ratio 3	Optimized Mix Ratio 4
Degree of fluidity	143	136	145	138	185	178	165	163

**Table 7:** Physical properties of the cement.

Standard Consistency Water Consumption (%)	Specific Surface Area (m^2^/kg)	Stability	Density (g/cm^3^)	Initial Setting Time (min)	Final Setting Time (min)
28.6	388	1	2.62	212	285

**Table 8:** Chemical composition of the cement.

Chemical Composition	MgO	SO_3_	CaO	Al_2_O_3_	Fe_2_O_3_	SiO_2_	Ignition Loss
Cement	4.26	2.37	60.31	5.14	3.02	20.13	4.77

**Table 9:** Technical index of fly ash.

Ignition Loss (%)	Moisture Content (%)	SO_3_ (%)	CaO (%)
2.20	0.20	1.70	0.10

**Table 10:** Technical index of silica fume.

Activity Index (%)	SiO_2_ (%)	Ignition Loss (%)
108	94.8	3.28

**Table 11:** Technical index of slag powder.

Density (g/cm^3^)	SO_3_ (%)	Cl (%)	Glass Content (%)	Ignition Loss (%)
7.55	3.47	0.02	91.6	0.62

**Table 12:** Standard sand technical indicators.

Soil Content (%)	Density (g/cm^3^)	Ignition Loss (%)	SiO_2_ (%)
0.10	2.67	0.23	99.6

**Table 13:** Standard sand gradation (mm).

>2.0	2.0~1.0	1.0~0.5	0.5~0.15	0.15~0.075	<0.075	Summation
0	33	34	20	12	1	100

**Table 14:** Quartz sand technical indicators.

SiO_2_ (%)	Cl (%)	Sulfide and Sulfate (%)	Mica (%)
98.8	0.01	0.21	0.02

**Table 15:** Quartz sand gradation.

Particle Size Requirements (mm)	1.25~0.63 mm		0.63~0.315 mm	
	≥1.25	<0.63	≥0.63	<0.315
Super-size particle content (%)	2	5	3	4

**Table 16:** Measurement Results of the Electric Flux Values of UHPC with Different Curing Temperatures.

Peer Group	Electric Flux (C)	The Converted Electric Flux (C)
1-1	217.00	195.84
1-2	100.96	91.12
2-1	202.73	182.96
2-2	91.72	82.78
3-1	146.37	132.10
3-2	73.27	66.13
4-1	139.59	125.98
4-2	62.41	56.32

Note: -1 is the standard curing condition; -2 is high temperature curing condition.

**Table 17:** Anti-chloride ion erosion grade of concrete.

Electric Conduction Quantity (C)	>4000	2000~4000	1000~2000	100~1000	<100
Chloride ion permeability	High	Middle	low	extreme low	Neglect

**Table 18:** Concrete mass change rate at different curing temperatures (%).

The Number of Freeze-Thaw Cycles	Standard Maintenance (%)				High Temperature Curing (%)			
	1	2	3	4	1	2	3	4
100	0.11	0.09	0.05	0.04	0.09	0.06	0.02	0.02
200	0.24	0.11	0.12	0.15	0.16	0.1	0.09	0.1
300	0.28	0.16	0.19	0.21	0.22	0.17	0.13	0.17
400	0.37	0.32	0.27	0.27	0.29	0.29	0.2	0.22
500	0.64	0.52	0.43	0.39	0.41	0.43	0.32	0.28

**Table 19:** Determination results of concrete compressive strength under different freeze-thaw cycles.

Peer Group	Initial Strength (MPa)	500 Freeze-Thaw Cycles (MPa)	Compressive Strength Loss Rate (%)
1-1	120.8	110.5	8.53
1-2	124.3	116.9	5.95
2-1	116.5	109.3	6.18
2-2	122.6	116.8	4.73
3-1	122.5	115.7	5.55
3-2	130.8	126.4	3.36
4-1	123.8	118.5	4.28
4-2	139.6	135.7	2.79

## Data Availability

All data generated or analysed during this study are included in this published article.

## Abbreviations

The following abbreviations are used in this manuscript:

UHPC	Ultra-high performance concrete
W/B	Water-binder ratio
AFt	Hydrated calcium aluminate sulfate

## References

[1] Pierrehumbert R. (2019). There is no Plan B for dealing with the climate crisis. Bull. At. Sci..

[2] Yin F., Mei S. (2025). ‘China Maritime Economic Statistics Bulletin 2024’ was released. Waterw. Port.

[3] Mehta P.K., Monteiro P.J.M. (2014). Concrete: Microstructure, Properties, and Materials.

[4] Scrivener K., John V.M., Gartner E. (2018). Eco-efficient cements: Potential economically viable solutions for a low-CO_2_ cement-based materials industry. Cem. Concr. Res..

[5] Richard P., Cheyrezy M. (1995). Composition of reactive powder concretes. Cem. Concr. Res..

[6] Graybeal B.A. (2006). Material Property Characterization of Ultra-High Performance Concrete (FHWA-HRT-06-103).

[7] Sanjuán M.Á., Andrade C. (2021). Reactive Powder Concrete: Durability and Applications. Appl. Sci..

[8] Herki A.M.B. (2024). Strength and Absorption Study on Eco-Efficient Concrete Using Recycled Powders as Mineral Admixtures under Various Curing Conditions. Recycling.

[9] Sun J., Zhang C., Mao J., Li M., Gao X. (2024). Mechanism of influence of curing system on strength of ultra-high performance concrete. Material Guide.

[10] Mo L., Wei L., Zhou Q., Yu L. (2024). Compression freeze-thaw damage evolution of fly ash concrete under different curing systems. Hydropower Energy Sci..

[11] Davraz M., Isildar N., Kaplan A.N. (2025). Investigation of the effects of steam curing of concrete at different temperatures on cost and compressive strength by response surface methodology. Constr. Build. Mater..

[12] Sudarsono I., Wahyudi S.I., Pratiwi A.H. (2025). Concrete Durability with Fly Ash Blended Cement for Coastal Construction. BIO Web Conf..

[13] Maohua Z., Ronghua X., Ke L., Yunbo L., Weiguo S. (2022). Research Progress on Durability of Marine Concrete under the Combined Action of Cl^−^ Erosion, Carbonation, and Dry–Wet Cycles. Rev. Adv. Mater. Sci..

[14] Raj Kishore G.V.V.K. (2021). Experimental Study on Strength Attainment of Concrete Containing Silica Fume and Fly Ash. IOP Conf. Ser. Mater. Sci. Eng..

[15] Wang X., Dong Y. (2019). Study on Sulfate Attack Resistance of Concrete with Different Mineral Admixtures. Yangtze River.

[16] Zhao Y., Zhang Z., Wang P., Zhang M. (2022). Influence of Mineral Admixtures on the Properties of UHPC. Bull. Chin. Ceram. Soc..

[17] Singh R., Haq M., Khan A.R. (2024). Influence of Industrial Waste and Mineral Admixtures on Durability and Sustainability of High-Performance Concrete. Environ. Sci. Pollut. Res. Int..

[18] Gu Y., Duan Y., Guan L., Li Z. (2025). Research Progress on the Effect of Mineral Admixtures on the Frost Resistance of Concrete. Low Temp. Build. Technol..

[19] Abbas S., Khan M.I. (2019). Durability of ultra-high performance concrete: A review. Constr. Build. Mater..

[20] Ghafari E., Costa H., Júlio E. (2015). Effect of nano-silica addition on durability of UHPC. Constr. Build. Mater..

[21] Zhang M., Li Z., Du L., Tian Z., Liu D. (2024). Durability of marine concrete with nanoparticles under coupled action of fatigue load, dry–wet cycles and Cl^−^ corrosion. Mag. Concr. Res..

[22] Zhang M., Xu R., Tian Z., Li Z. (2023). Durability of Marine Concrete with Nanoparticles under the Joint Effect of Dry–Wet Cycles, Cl^−^ Erosion, and Carbonation. J. Mater. Civ. Eng..

[23] Zhang M., Du L., Li Z., Xu R. (2023). Durability of marine concrete doped with nanoparticles under joint action of Cl- erosion and carbonation. Case Stud. Constr. Mater..

[24] Sun T., Wang X., Ashour A., Ding S., Li L., Han B. (2025). High-durability, low-carbon, and low-cost nano-engineered concrete for marine concrete infrastructures. Cem. Concr. Compos..

[25] Zhao J., Huang L., Xie J. (2019). Effects of preparation process on the workability and compressive strength of ultra-high performance concrete. Concrete.

[26] Shi C., Qiu W. (2021). Gradation Design and Optimal Dosage of Quartz Sand in Ultra-High Performance Concrete. Subgrade Eng..

[27] (2015). Reactive Powder Concrete (RPC).

[28] (2018). Basic Properties and Test Methods of Ultra-High Performance Concrete (UHPC).

[29] Men G., Jia X., Zhu W. (2023). Study on influencing factors of workability and mechanical properties of ultra-high performance concrete. Concr. Cem. Prod..

[30] Qing T., Li X., Zhao J., Chen Z., Lu Z., Qi M., Li J. (2023). Preparation and performance study of high titanium slag UHPC slurry. Concr. Cem. Prod..

[31] Shang X. (2022). Study on the Influence of Quartz Sand Gradation on the Performance of Ultra-High Performance Concrete. Master’s Thesis.

[32] (2005). Determination Method of Fluidity of Cement Mortar.

[33] Khan W.A., Kumar S. (2024). Development of Rheology in the Area of High-Performance Concrete—A Review. J. Appl. Eng. Sci..

[34] Benaicha M., Jalbaud O., Roguiez X., ALaoui A.H., Burtschell Y. (2025). Exploring rheological properties of self-compacting concrete: Mineral and chemical admixture impacts. Arch. Civ. Mech. Eng..

[35] (2009). Standard Test Method for Long-Term Performance and Durability of Ordinary Concrete.

